# Gallbladder Volvulus: A Great Mimicker of Acute Cholecystitis

**DOI:** 10.7759/cureus.101934

**Published:** 2026-01-20

**Authors:** Fallon M Cassidy, Joseph R Peterson, Hugo C Araujo Lino

**Affiliations:** 1 College of Osteopathic Medicine, Pacific Northwest University Health Sciences, Yakima, USA; 2 General Surgery, Saint Alphonsus, Nampa, USA

**Keywords:** acute acalculous cholecystitis (aac), biliary diseases, gallbladder imaging modalities, gallbladder volvulus, laparoscopic cholecystectomy (lc)

## Abstract

Gallbladder volvulus is a rare disease process, with approximately 500 reported cases in the current literature. Preoperative diagnosis is challenging, with only about 80 cases reported as being diagnosed prior to surgery, as it is frequently misdiagnosed as acute cholecystitis. Prompt recognition is vital, as the mainstay of treatment is emergent surgical intervention.

This case describes an 84-year-old female who presented with right upper quadrant abdominal pain. Initial computed tomography (CT) demonstrated biliary anatomic abnormalities with relatively unremarkable laboratory values, and a presumptive diagnosis of biliary colic was made, concerning for the development of acute cholecystitis. The patient was discharged with plans for an outpatient ultrasound and follow-up, but returned the following day with worsening symptoms. Repeat imaging demonstrated gallbladder wall thickening without cholelithiasis. Laparoscopic cholecystectomy was performed, and intraoperative findings confirmed gallbladder volvulus. The patient had an uneventful postoperative course.

Early recognition of gallbladder volvulus may improve outcomes and prevent complications. Characteristic imaging findings and atypical clinical presentations should raise suspicion for gallbladder volvulus and prompt further investigation. Although rare, gallbladder volvulus should be considered in the differential diagnosis of pediatric and elderly female patients presenting with symptoms similar to acute cholecystitis.

## Introduction

Gallbladder volvulus occurs when the gallbladder twists around the cystic pedicle, which contains the cystic duct and cystic vessels (artery and vein), causing either a partial torsion (<180°) or complete torsion (≥180°). This twist obstructs the cystic duct and vessels, impairing vascular flow to the gallbladder wall and leading to acute inflammation and ischemia, with subsequent necrosis if left unresolved [1,2]. Unlike acute calculous cholecystitis, which is an inflammatory process secondary to stone-induced biliary obstruction, gallbladder volvulus is caused by a distinct anatomic abnormality resulting in ischemia. Both conditions may require surgical intervention if conservative management fails; however, gallbladder volvulus necessitates more urgent surgery due to compromise of the gallbladder’s blood supply. Often, the gallbladder is completely detached from its usual position in the gallbladder fossa; in some cases, it may even be located retrohepatically [1]. This abnormal mobility could increase the technical difficulty of cholecystectomy.

The diagnosis of gallbladder volvulus is exceptionally uncommon, with an average incidence of one in 365,000 cases of hospitalized patients secondary to gallbladder disease. This data is based on historical case series rather than population-level epidemiologic data [1]. Gallbladder volvulus most commonly occurs in elderly women in the sixth to eighth decade of life and has not been found to be associated with any known genetic abnormalities, with the etiology commonly attributed to a combination of anatomical variants and age-related changes [1,3]. These include an increased length of mesentery and increased mesenteric laxity, which collectively permit excessive gallbladder motility, therefore predisposing to torsion [3]. Other risk factors include the surrounding bowel’s peristaltic motion and abnormalities in the spinal column, such as kyphoscoliosis [3,4]. 

Gallbladder volvulus is rarely diagnosed preoperatively, as its clinical presentation closely mimics the more common diagnosis of acute cholecystitis, with only a limited number of cases reported as being identified prior to surgery in the current literature [5]. There are imaging findings on CT, including "whirl sign" and ultrasound findings such as cystic duct knot sign that can help indicate gallbladder volvulus [6,7]. This case report aims to highlight the diagnostic challenges associated with gallbladder volvulus, describe imaging findings that are more specific to this entity, and discuss the clinical features in this case that may help distinguish gallbladder volvulus from more typical gallbladder pathology.

## Case presentation

An 84-year-old woman with a significant past medical history of heart failure with preserved ejection fraction, ascending aortic dilation, hypertension, coronary artery calcification, and chronic hypoxic respiratory failure on as-needed supplemental oxygen initially presented with one day of constant pressure-like pain in the right upper quadrant and lower chest. The pain was sudden in onset and began while she was performing physical labor on the farm. The pain radiated to the epigastric area and was associated with nausea and dry heaving. Vital signs at that time were as follows: blood pressure, 155/97 mmHg; heart rate, 92 bpm; respiratory rate, 20 breaths per minute; temperature, 36.7℃ (98.1℉); and oxygen saturation, 97% on baseline supplemental oxygen (4 L via nasal cannula). Pertinent physical exam findings included diffuse upper abdominal discomfort with no focal tenderness and a negative Murphy's sign. Laboratory tests showed total bilirubin, alkaline phosphatase, C-reactive Protein (CRP), white blood cell (WBC) count, blood urea nitrogen (BUN), creatinine (Cr), and lactic acid were all within normal limits. Additionally, the patient's cardiac evaluation was found to be negative.

**Figure 1:**
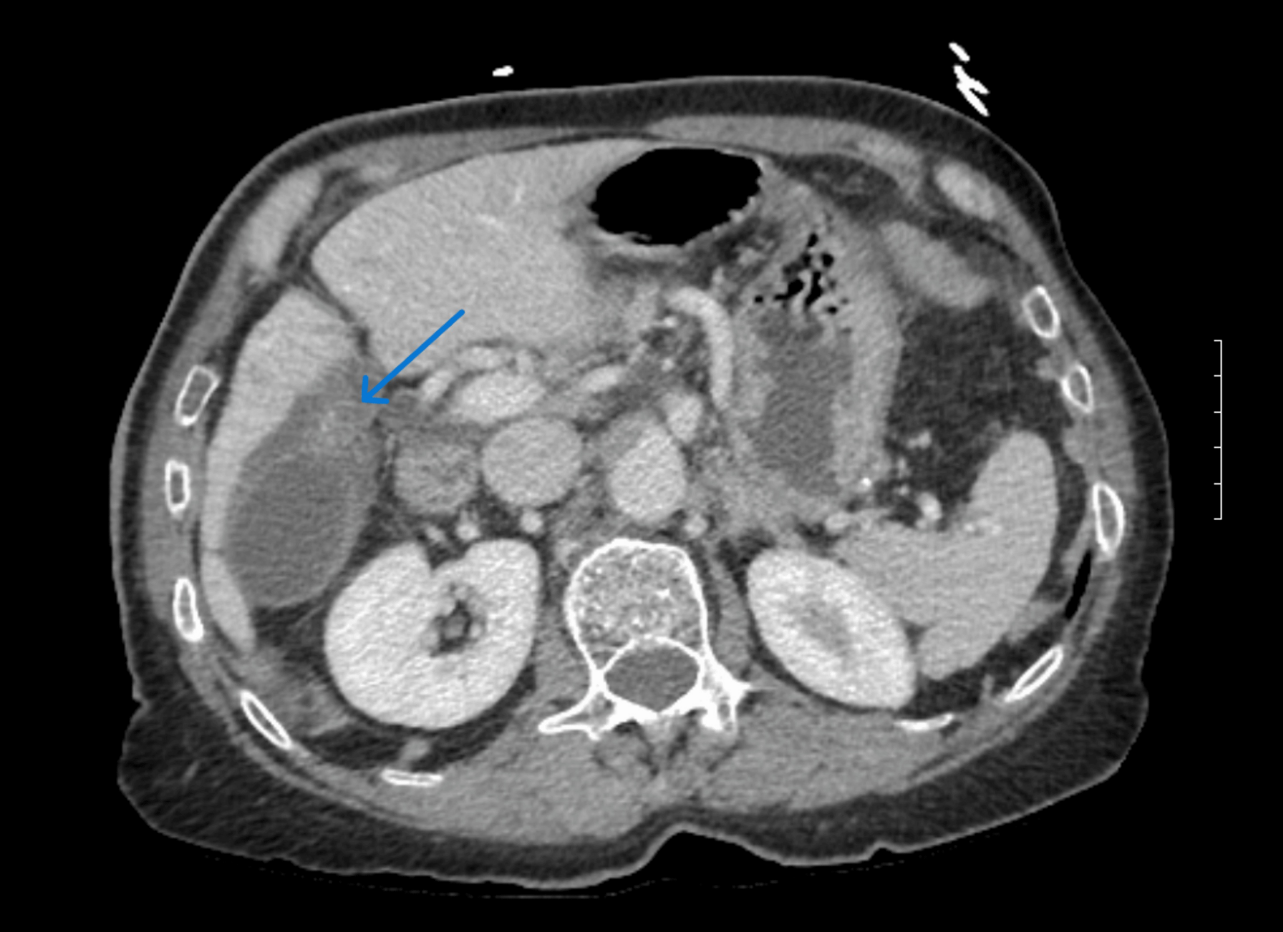
CT Image of gallbladder CT image showing torsion of the gallbladder around the cystic pedicle with dilation of the gallbladder distally. The blue arrow indicates the early appearance of the "whirl sign" as seen by the two radiopaque conical shaped linear swirls.

Imaging consisted of a computed tomography (CT) scan, which demonstrated gallbladder wall thickening and edema, which could represent acute cholecystitis; however, there was no presence of pericholecystic fluid or fat stranding at that time. Additionally, the initial CT scan interpretation was significant for mild diffuse intrahepatic biliary ductal dilatation and mild distal common bile duct dilation for age. These findings appeared related to tortuosity secondary to asymmetric elevation of the right hemidiaphragm and distortion of right upper quadrant structures. The radiology report was determined to be nonspecific, as it did not explicitly state that the findings listed above were secondary to gallbladder volvulus. Upon further review of the image, a finding more specific to gallbladder volvulus was identified: an early “whirl sign” (Figure 1).

She was subsequently discharged from the emergency department (ED), with a mild initial presentation and the ability to tolerate liquids. For pain management, she was given hydrocodone-acetaminophen (Norco®) and ondansetron (Zofran®). Considering CT imaging concerning inflammation of the gallbladder; however, no signs of acute infection, she was instructed to schedule an urgent outpatient cholecystectomy and undergo a follow-up ultrasound for further evaluation of the gallbladder.

After discharge, the patient’s pain persisted. She presented to a different ED the following day with worsening abdominal pain and one episode of bilious emesis. She was transferred to our facility with a presumptive diagnosis of acute cholecystitis. On examination, she had mild respiratory distress, a soft abdomen with normal bowel sounds, and focal peritonitis given right upper quadrant tenderness. Total bilirubin had increased to 2.1 mg/dL (reference range, 0.1-1.2 mg/dL), but alkaline phosphatase, aspartate aminotransferase (AST), alanine aminotransferase (ALT), WBC count, and CRP were all within normal limits. Abdominal ultrasound showed gallbladder wall thickening with a small volume of pericholecystic fluid, no cholelithiasis, and a positive sonographic Murphy’s sign. The maximum common bile duct diameter measured 4.2 mm. There was no impression reported regarding mobility or abnormal positioning of the gallbladder. 

**Figure 2:**
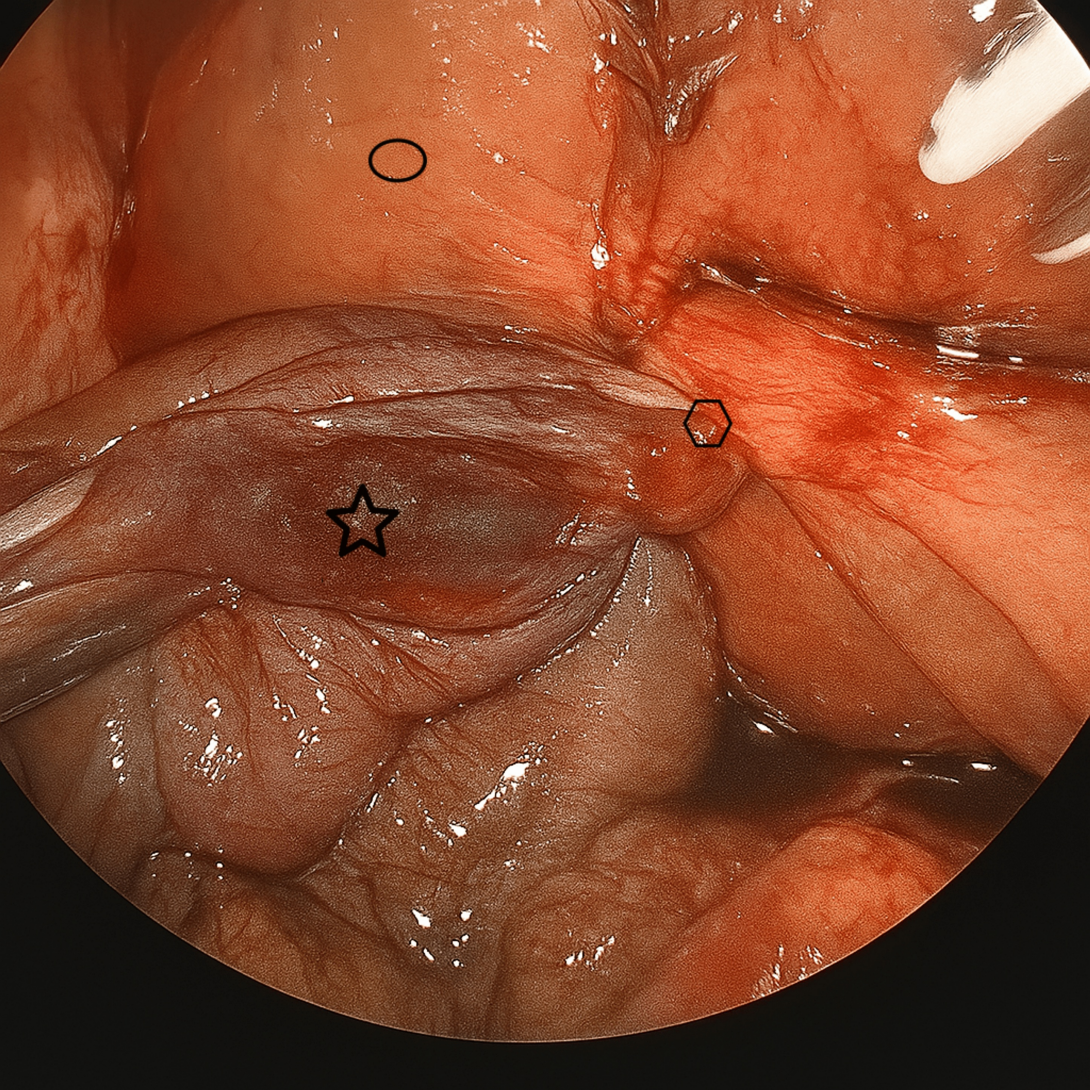
Intraoperative photo of the gallbladder volvulus Intraoperative laparoscopic view of the gallbladder volvulus. The gallbladder (star) was laparoscopically detorsed, with the image demonstrating only partial torsion around the gallbladder pedicle (polygon) containing the cystic duct and the cystic artery. The liver (oval) is shown for anatomical reference.

**Figure 3:**
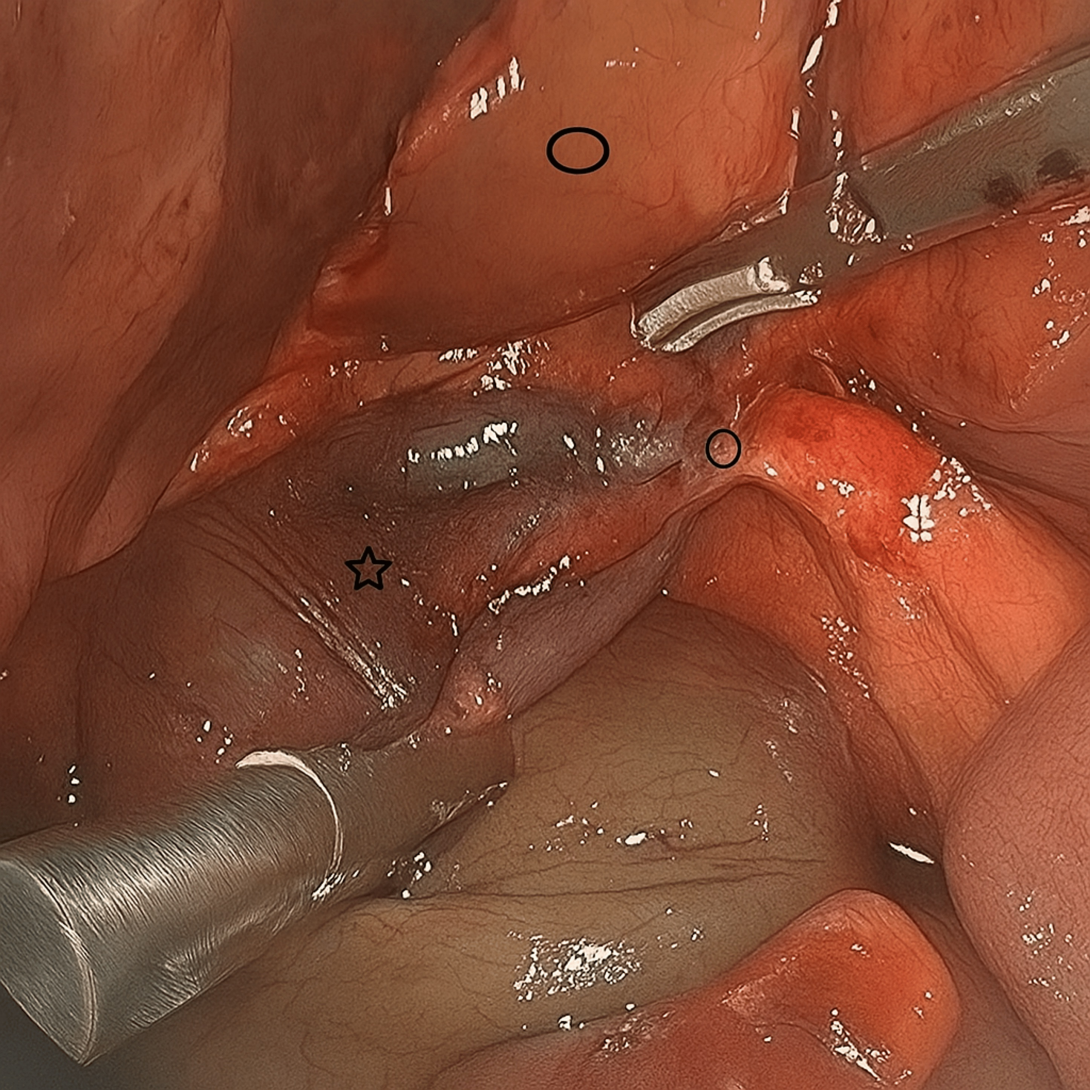
Intraoperative photo of gallbladder volvulus and long cystic pedicle following detorsion Intraoperative laparoscopic view of the gallbladder following complete detorsion. The star indicates the gallbladder, the circle highlights the lengthened cystic pedicle following careful intraoperative unwinding, and lastly the oval denotes the liver as an anatomical reference point.

The patient was treated with intravenous fluids, antibiotics, and analgesics. She underwent a laparoscopic cholecystectomy the following day. Intraoperatively, a distended gallbladder was noted with greater than 360-degree counterclockwise torsion of the cystic duct and artery. The gallbladder was displaced from its anatomic position within the gallbladder fossa, with its only attachment to the liver being the twisted cystic duct and artery (Figures 2, 3). 

During this case, the gallbladder was manipulated, and indocyanine green (ICG) was used to better determine surgical anatomy. An intraoperative cholangiogram was performed and demonstrated the cystic duct joining the common bile duct, with contrast entering the duodenum. Following confirmation of duct patency, subsequent ligation of the cystic vessels and ducts took place. There was mild intraoperative evidence of gallbladder necrosis near the neck of the gallbladder. The gallbladder was subsequently removed through one of the laparoscopic port sites. Following this, a subhepatic drain was placed. The postoperative course was uneventful, with complete resolution of the pain and emesis. The patient was discharged on postoperative day two.

## Discussion

This case illustrates the diagnostic challenge of a patient presenting with severe, self-reported 9/10 abdominal pain with minimal corroborating laboratory and imaging abnormalities. The patient's mild initial presentation led to a missed opportunity, as although the CT did not evidence acute cholecystitis, it did demonstrate the nonspecific presence of biliary tortuosity. This potentially meaningful finding should have prompted further evaluation with additional diagnostic workup, underscoring the difficulty in the diagnosis of gallbladder volvulus. 

Gallbladder volvulus is thought to result from the gallbladder rotating around the cystic pedicle, causing torsion and obstruction of the cystic duct, thereby impeding vascular flow to the gallbladder wall [1]. This case illustrates complete torsion, with greater than 360° of rotation, compared to an incomplete torsion, which involves a rotation less than 180° [7]. The torsion may occur in either a clockwise or counterclockwise direction. In this case, the causality behind the rotation has not yet been determined; we hypothesize that physical activity may have contributed to this torsion, given the patient's increased physical exertion when the sudden onset of right upper quadrant pain began. This potential trigger, involving increased intrabdominal pressure or exertional forces leading to an increase in gallbladder mobility, has not been well characterized in the existing literature. Other theories that have been characterized include visceral peristalsis - it has been hypothesized that clockwise rotation is caused by gastric peristalsis, whereas counterclockwise rotation is hypothesized to be attributed to peristalsis of the transverse colon; however, this continues to be theoretical in nature [8]. Although this disease has a bimodal age distribution, with most patients between 60 and 80 years old, and a smaller peak in the pediatric population, it continues to be a rare diagnosis amongst all age groups. Among adults, there is a disproportionate number of females affected, with a female-to-male ratio of approximately 4.5:1, whereas in children the ratio is reversed, at approximately 2.5:1 [9,10]. It is important to recognize the epidemiological patterns, as patient presentations that are atypical may further magnify the delay in recognition and surgical treatment. 

Preoperative diagnosis of gallbladder volvulus remains challenging. Only a few cases have been documented as diagnosed prior to surgery, with most discovered intraoperatively [5]. The cystic duct knot sign can be identified on ultrasound or CT and appears as a stretched cystic duct with a hyperechoic, funnel-shaped nodule and multiple linear echoes coalescing toward the tip near the neck of the gallbladder, representing the torsed cystic duct [11,12]. The whirl sign on non-contrast CT imaging may also indicate torsion, appearing as a dense nodule near the cystic pedicle with twisting of adjacent fat [2]. Additionally, a floating gallbladder may be visualized on ultrasound, showing a change in orientation from vertical to horizontal with associated wall thickening. On hepatobiliary iminodiacetic acid (HIDA) scan, a “bullseye” appearance may be seen due to radiotracer accumulation medial to the gallbladder within the common bile duct; there is no uptake of dye in the gallbladder itself because of cystic duct occlusion, despite the absence of gallstones [1,13,14].

In contrast, acute cholecystitis typically demonstrates gallbladder wall thickening, pericholecystic fluid, a positive sonographic Murphy’s sign, biliary sludge, and, most commonly, the presence of gallstones, often paralleled by an inflammatory response. Gallbladder volvulus is a rare entity that presents with symptoms strikingly similar to acute cholecystitis; however, can be discriminated from acute cholecystitis by the clinical progression of the patient as well as the discordance between the patient's reported severity and clinical laboratory findings. This was demonstrated in our case, with the patient reporting severe abdominal pain refractory to medication despite only initial mild abnormal laboratory values. This discrepancy is hypothesized from the cystic duct demonstrating an incomplete obstruction during the initial twisting, resulting in normal bilirubin levels and liver enzymes such as alanine aminotransferase (ALT) and aspartate aminotransferase (AST) [13]. A patient presenting with gallbladder volvulus will often have pain unresponsive to conservative management, with rapid progression of symptoms, underscoring the vascular compromise in gallbladder volvulus, as compared to the inflammatory obstruction seen with acute cholecystitis.

Awareness of the clinical progression and laboratory findings is essential for prompt recognition and reassessment of the diagnosis, as gallbladder volvulus is a rare entity that can lead to an increased risk of anchoring bias. Although both conditions share overlapping clinical features, gallbladder volvulus carries a significantly higher mortality rate, approximately 6% compared to 3.6% for acute calculous cholecystitis [10]. However, the statistical significance should be interpreted with caution, given the lack of direct comparison. The mortality estimates for gallbladder volvulus are derived from limited case series, in contrast to the population-based data derived from a robust cohort of individuals with acute calculous cholecystitis. Nonetheless, this potential for increased mortality highlights the importance of remaining vigilant against anchoring bias, especially when a presentation of acute calculous cholecystitis deviates from the expected clinical course. 

A literature review by Reilly et al. found seven reported deaths from gallbladder volvulus within the past two decades, none of which occurred in patients diagnosed preoperatively [9]. Of the approximately 500 total reported cases, 80 were diagnosed preoperatively, and all of those 80 patients survived [8]. These findings underscore the importance of early recognition and timely surgical management. There is considerable variability in patient comorbidities when estimating mortality rates, and additional research is warranted to examine pre-surgical medical factors and their association with postoperative outcomes following cholecystectomy.

The definitive treatment for gallbladder volvulus is cholecystectomy, either laparoscopic or open, with careful detorsion and dissection to expose the cystic duct and artery while avoiding injury to surrounding structures [1]. A few cases have reported attempted detorsion via endoscopic retrograde cholangiopancreatography (ERCP); however, this approach is not routinely recommended, as it may predispose patients to recurrent volvulus and irreversible ischemic damage to the gallbladder [8]. Untreated gallbladder volvulus can result in serious complications, including infarction, necrosis, gangrene, perforation, biliary peritonitis, multiorgan failure, and death [1].

This case is notable in that the initial CT scan suggested a nonspecific finding of gallbladder tortuosity, yet the patient was discharged home, given her mild initial presentation and lack of significant lab abnormalities, with likely bias anchoring toward presumptive acute cholecystitis, given the typical presentation of right upper quadrant abdominal pain. It highlights the need for increased awareness of gallbladder volvulus as a rare but potentially fatal condition that can easily be mistaken for more common biliary pathologies. By contributing to the growing body of literature, this case emphasizes the importance of understanding the epidemiology and variable presentations of gallbladder volvulus. Although the patient in this report experienced a favorable outcome, missed diagnoses can be life-threatening, reinforcing the necessity for heightened clinical vigilance. Furthermore, this case underscores the importance of maintaining a lower threshold for additional imaging and comprehensive evaluation in elderly women presenting with right upper quadrant pain. Ultimately, strengthening clinical suspicion and refining diagnostic approaches can significantly improve patient outcomes.

## Conclusions

In an elderly female patient presenting with biliary symptoms, including right upper quadrant abdominal pain that is disproportionate to clinical findings and refractory to conservative management, early consideration of gallbladder volvulus is warranted with the obtainment of further diagnostic imaging modalities. To aid in preoperative identification and to guide the urgency of surgical intervention, a CT scan should be obtained, which may demonstrate classic findings such as a “floating gallbladder” or the “cystic duct knot sign.” Early recognition with the establishment of a preoperative diagnosis of gallbladder volvulus has important implications for emergency department evaluation and surgical triage, as rapid recognition can reduce the time from admission to surgery, improve surgical outcomes, and minimize the risk of tissue necrosis and gangrene.
